# Perceptions of Skills Needed for STEM Jobs: Links to Academic Self-Concepts, Job Interests, Job Gender Stereotypes, and Spatial Ability in Young Adults

**DOI:** 10.3390/jintelligence12070063

**Published:** 2024-06-27

**Authors:** Margaret L. Signorella, Lynn S. Liben

**Affiliations:** 1 Department of Psychology, The Pennsylvania State University, Brandywine Campus, 25 Yearsley Mill Road, Media, PA 19063, USA; 2 Department of Psychology, The Pennsylvania State University, University Park, PA 16802, USA; liben@psu.edu

**Keywords:** STEM, spatial skills, job interests, academic self-concepts, O*NET, job skills, gender stereotypes, situated expectancy value theory (SEVT)

## Abstract

Gender gaps in spatial skills—a domain relevant to STEM jobs—have been hypothesized to contribute to women’s underrepresentation in STEM fields. To study emerging adults’ beliefs about skill sets and jobs, we asked college students (*N* = 300) about the relevance of spatial, mathematical, science and verbal skills for each of 82 jobs. Analyses of responses revealed four job clusters—quantitative, basic & applied science, spatial, and verbal. Students’ ratings of individual jobs and job clusters were similar to judgments of professional job analysts (O*NET). Both groups connected STEM jobs to science, math, and spatial skills. To investigate whether students’ interests in STEM and other jobs are related to their own self-concepts, beliefs about jobs, and spatial performance, we asked students in another sample (*N* = 292) to rate their self-concepts in various academic domains, rate personal interest in each of the 82 jobs, judge cultural gender stereotypes of those jobs, and complete a spatial task. Consistent with prior research, jobs judged to draw on math, science, or spatial skills were rated as more strongly culturally stereotyped for men than women; jobs judged to draw on verbal skills were more strongly culturally stereotyped for women than men. Structural equation modeling showed that for both women and men, spatial task scores directly (and indirectly through spatial self-concept) related to greater interest in the job cluster closest to the one O*NET labeled “STEM”. Findings suggest that pre-college interventions that improve spatial skills might be effective for increasing spatial self-concepts and the pursuit of STEM careers among students from traditionally under-represented groups, including women.

## 1. Introduction

The underrepresentation of women in STEM (science, technology, engineering, and mathematics) careers in the United States continues to be evident in recent data analyzed by the U.S. Department of Labor Women’s Bureau (2021) from census data and by the National Science Foundation’s National Center for Science and Engineering Statistics (NCSES 2023). These gaps are concerning to U.S. educators and science advocates (e.g., Liben and Coyle 2014; Varma 2018; Xie et al. 2015). Importantly, many of the occupations that continue to show very low percentages of women are the very same occupations that have been projected by the U.S. Bureau of Labor Statistics Occupational Outlook Handbook (U.S. Bureau of Labor Statistics 2023) as growing faster than average in the United States (e.g., computer-related fields, architecture, and engineering). MacLean (2017) argued that many undesirable consequences result from the lack of diversity in U.S. STEM occupations. There are impacts on the economy when skilled jobs are not filled, on the types of solutions that technology can offer when only a subset of humans is designing and implementing technology solutions, and on members of minoritized groups when those individuals are not acquiring satisfying and high-paying jobs.

### 1.1. STEM Careers and Spatial Skills

Gender gaps in spatial skills have been proposed as one possible barrier to women entering STEM careers (Baker and Cornelson 2018). Overall, women and girls score lower on various tests of spatial skills than men and boys (e.g., Reilly et al. 2017) and there is clear evidence that spatial skills are correlated with STEM success (e.g., Kell and Lubinski 2013; Khine 2017; Wai et al. 2009). Much of the research on spatial skills focuses on methods for improving spatial performance (e.g., Uttal et al. 2013) and the links between spatial performance and math skills (e.g., Mix et al. 2016). Many intervention studies have targeted engineering majors to determine if training in spatial skills can aid in student retention and later career success (e.g., Sorby et al. 2013).

One reason considerable attention has been directed to the impact of spatial skill interventions in experimental research and engineering programs is the recognition that traditional K-12 curricula do not explicitly target spatial skills (e.g., Liben 2006; National Research Council 2006; Newcombe 2013). That is, although spatial skills are relevant in some standard school courses (e.g., geometry and drafting), there is little explicit focus on spatial thinking and skills even in these courses, leading some educators to plead for more spatial-focused instruction (e.g., see Budai 2013; Nagy-Kondor 2017; Olkun 2003). The arts offer another possible avenue for developing spatial skills, but arts courses and programs have diminished in formal education as the emphasis on standardized tests in other school domains has increased (Rabkin and Hedberg 2011). Kell and Lubinski (2013) further argue that the neglect of spatial skills extends beyond K-12 to college entrance exams and the occupational sphere. The paucity of school-based spatial education heightens the importance of informal means for spatial learning, such as playing with stacking and interlocking blocks, using construction toys, or playing with toy vehicles. Higher levels of engagement in experiences like these—more common among boys and men than girls and women—are correlated with better performance on spatial tests (e.g., Doyle et al. 2012).

More generally, research bearing on one of the major theories of career development—social cognitive career theory (SCCT; e.g., Lent and Brown 2019)—has shown that prior experiences predict crucial perceptions of self-efficacy and expectations of success (e.g., Schaub and Tokar 2005). For example, consider the Learning Experiences Questionnaire (LEQ; Schaub 2003, pp. 198–204), which has been used to assess prior career-related experiences. Many LEQ items share a methodology with spatial activity inventories in aiming to assess exposure outside the classroom to pursuits that might be career-related (e.g., “I have made repairs around the house”), although none of the 120 items on the LEQ pertains explicitly to spatial abilities. Gender differences have also been observed in studies of other kinds of STEM-related learning experiences. For example, boys and men report having had more investigative experiences, such as scientific activities, and more exposure to scientists (Jones et al. 2021; Tokar et al. 2007; Williams and Subich 2006) compared with girls and women.

A crucial area that needs more attention is whether young students’ perceptions of their own spatial abilities affect their career choices. Girls and women view their spatial skills less positively than boys and men (Matthews et al. 2024). Given that there are, in fact, consistent gender differences favoring men and boys in some measures of spatial skills, these gender differences in confidence or skill ratings are expected. However, Ariel et al. (2018) concluded from their data that women consistently underestimated their performance on spatial tests, irrespective of whether there were actual gender differences favoring men on a particular test. Similarly, Estes and Felker (2012) and Arrighi and Hausmann (2022) found that self-confidence and spatial anxiety were mediators of gender differences in mental rotation. Career development theories clearly include self-confidence and self-efficacy as crucial components, but missing is career-related research focused specifically on (a) self-confidence and self-efficacy within the domain of spatial skills and (b) whether these self-focused constructs about spatial skills are implicated in young adults’ pursuit of STEM careers.

### 1.2. Gender Stereotyping

Another factor that may deter women from STEM careers is that many STEM careers have long been viewed as stereotyped for men. For example, Cheryan et al. (2015) examined views of computer scientists and engineers, both of which are STEM careers that continue to show gender and racial disparities favoring men and White individuals (e.g., MacLean 2017; NCSES 2023). Cheryan et al. concluded that computer scientists and engineers are viewed by students as socially awkward, obsessed with technology, and masculine in characteristics or interests. Another characteristic commonly associated with STEM is intelligence. This association is also relevant to the STEM gender gap, as research has shown that young children associate high intelligence with boys and men more strongly than with girls and women (Bian et al. 2017, 2018). Also demonstrating the relevance of career-related gender stereotypes are studies showing that children and adolescents endorse the stereotype that girls are less interested than boys in computer science or engineering and that girls who endorse this stereotype are less interested in those careers (Master et al. 2021). It is important, therefore, to examine beliefs about cultural gender stereotypes of job interests.

The contributions of gender stereotyping and gender role expectations to career choice and achievement motivation are predicted in SCCT (e.g., Tokar et al. 2007) and in situated expectancy value theory (SEVT; Eccles and Wigfield 2020). Both theories conceptualize gender stereotypes as potential precursors to the development of achievement- and career-related self-concepts. More specifically, SEVT leads to the expectation that individuals’ job choices will be affected, first, by their own endorsements of their culture’s gender role expectations and stereotypes and, second, by their beliefs about their own job-relevant skills. From this perspective, women would be more likely to follow educational and career paths toward STEM under several conditions, specifically if they believe themselves to have strong skills in relevant domains (e.g., strong spatial skills); if they do not view or accept the STEM domains as exclusively masculine; and if they see value and obtain personal satisfaction from pursuing relevant activities and outcomes (e.g., they enjoy spatial challenges and achievements such as those entailed in designing or constructing buildings or equipment).

### 1.3. The Present Research

Prior research has already established that individuals’ self-conceptions and gender stereotyping of academic domains and jobs are linked to career choices (e.g., Wang and Degol 2017). We do not yet know, however, if young adults—particularly women—are avoiding STEM careers because they believe, first, that spatial skills are important for STEM jobs; second, that their own spatial skills are not adequate to meet the spatial skills needed for STEM jobs; and, third, that they would find it aversive, unappealing, or ineffective to try to improve their own spatial skills. If students are aware that spatial skills are important, then programs to improve women’s spatial performance would be advisable. If students do not already recognize that spatial skills are important, programs would be needed to help students see the value of such skills and to provide students with educational opportunities to develop those skills.

The first research question, therefore, was whether college students who are in the throes of making choices about academic majors and careers are aware of the importance of spatial skills in various occupations. To begin to address this question, we asked a sample of university students (*N* = 300) to rate each of 82 jobs on the degree to which each calls on spatial skills, and to rate the same jobs on the degree to which each calls on several other key academic skills—specifically, math, science, and English. We included math and science because both are central to STEM fields. We included English because although it is not viewed as a quintessential component of STEM, it is a skill domain commonly viewed by employers as being desirable in college graduates in general (e.g., Hirudayaraj et al. 2021) and because it provides a contrast to the STEM focus.

To identify jobs that are perceived to have similar skill demands, we first used a cluster analysis to group student ratings of needed job skills. Then, to learn if students’ perceptions are similar to the perceptions of expert job analysts, we compared the skill ratings made by student participants to occupational ratings made by U.S. labor force professionals as part of O*NET, created by the U.S. Department of Labor in 1999 (Mariani 1999). O*NET, which is in version 28 as of 2023, is designed to be “the nation’s primary source of occupational information” (NCOD 2023d). A National Research Council (2010) review concluded that O*NET is both “used and useful” (p. 1) and recommended that the U.S. Department of Labor continue the development and maintenance of the high-quality database that lies at the core of O*NET. O*NET offers worthwhile data for students, job seekers, and career counselors, providing information about job projections, potential salaries, and desired worker characteristics for specific jobs (Craven and Rivkin 2020).

The O*NET database, with its over 900 jobs, was an excellent benchmark against which to examine student perceptions because O*NET includes information about what skills are needed for most of the 82 jobs we had included in our survey. O*NET’s analysts rate the abilities required for each occupation using the O*NET categories grounded in the Fleishman and Reilly (1992) classifications and definitions (National Research Council 2010). According to Fleishman and Reilly, their classifications were derived from Fleishman’s own earlier work on ability organizational schemes, coupled with their collaborative review of research on empirical abilities employing factor analysis (see Fleishman and Reilly 1992, p. 2).

Two features of O*NET are particularly relevant to the present research. First, O*NET’s superordinate category of *cognitive abilities* includes the sub-group *spatial abilities*, which, in turn, contains the two sub-abilities of *spatial orientation* and *visualization* (NCOD 2023f). We describe these terms in more detail in the Methods. Second, as of 2023, O*NET included 291 jobs under the heading “STEM”, defined as jobs requiring “education in science, technology, engineering, and mathematics (STEM) disciplines” (NCOD 2023e). To test whether college students perceived job skills similarly to the assessments made by professional occupational analysts, we compared the obtained job skills clusters and the individual ratings made by students to the job judgments appearing on O*NET.

Our second research question was whether the students’ job skill groupings suggest barriers that might undermine women’s pursuit of STEM careers. With another sample of students (*N* = 292), we tested pathways derived from the extant gender-related research and literature, such as the Eccles and Wigfield (2020) SEVT model on achievement and the gender schema theories discussed by Martin et al. (2002). We chose measures that would permit us to examine associations among the groupings of job skill perceptions and gender differences in (a) job interests, (b) the culture’s gender stereotyping of the jobs, (c) self-concepts about academic skills (including STEM-related math, science, and spatial skills), and (d) performance on a spatial task. Given that the masculine stereotyping of STEM domains may be more impactful for women than men and thus result in different pathways, we tested whether the associations were different for women and men.

## 2. Materials and Methods

### 2.1. Participants

Students were recruited in 2011 from the psychology department participant pool at a public university campus in the mid-Atlantic region of the United States. The campus had over 40,000 undergraduates during the 2010–2011 academic year and a majority were in-state students, with roughly 72% receiving one or more forms of financial aid (National Center for Educational Statistics 2011b). Recruitment followed standard IRB and subject pool procedures in use at that time. These procedures included providing separate sign-up lists for women and men who wished to volunteer for research participation and informing participants they could skip questions they did not wish to answer. At the end of the survey, we requested demographic information from participants via questions about gender, race, age, and current academic unit.

Participants in one sample (*N* = 300) were asked to rate the degree to which they thought each of 82 jobs draws on four kinds of academic or cognitive skills. We refer in the current paper to these ratings as the **J**ob **S**kills **R**ating (JSR) survey, labeled online for participants as “What Do You Think? Job Skills”. Participants in the other sample (*N* = 292) were asked to rate their self-concepts in various academic domains, their interest in each of the 82 JSR jobs, and their beliefs about cultural gender stereotyping of those jobs. In addition, they were given a test to assess their spatial skill. We refer to this battery as the “**S**elf-**C**oncept, **I**nterest, **B**elief, and **S**patial” (SCIBS) survey; online, it was labeled as “What Do You Think?”.

The two resulting samples were demographically similar. Gender distributions were 49% women and 51% men in both samples; percentages of White students in the two samples were 89% and 86%, respectively, with Black or African American and Asian students the next most frequent; and the average age was 19 years in both samples. Participants in both samples came from every undergraduate academic unit on campus. The demographics of our samples were generally consistent with those of the university as a whole for the 2010–2011 academic year (National Center for Educational Statistics 2011a), although we note several modest differences. In particular, both our samples had higher percentages of women (49% in each compared to 45% in the university as a whole), relatively more White students (89% and 86% versus 76%), and relatively fewer Hispanic or Latino students (less than 1% in both samples versus 5%).

### 2.2. Materials and Procedure

#### 2.2.1. Selection of Jobs Included in Surveys

Given the focus of our research, a large proportion of the 82 jobs listed in the surveys were from one of the four STEM fields represented in the STEM acronym (i.e., science, technology, engineering, and math; NCSES 2023). Because many STEM jobs are traditionally viewed in the U.S. culture as masculine, the job list included a disproportionate number of jobs considered to be culturally “masculine”. About half the jobs (44) were taken from validated scales used to measure participants’ job interests and gender-stereotyped attitudes (Liben and Bigler 2002). We added 38 jobs (STEM and non-STEM). The additions were motivated by an effort to include jobs that call on an array of skills, including skills not generally associated with STEM (e.g., verbal skills), and jobs that call on varying levels of formal education.

#### 2.2.2. Measures Administered to the JSR Sample

Participants in the JSR sample were told they would complete a survey to help us “learn what skills people think different jobs require”. Four skills were named (math, spatial skills, English, and science) and each skill was explained briefly. Participants were asked to say how much they thought each of 82 jobs calls upon each of the named skills: *not at all, a little bit, some, pretty much,* or *a lot*. Responses were scored from 1 to 5, with higher numbers indicating stronger relevance of the named skill for the named job. Table 1 shows the skill descriptions provided, with one illustrative job included to show how each job was presented. Table 2 lists the 82 jobs included in the survey. In the surveys given to participants, jobs were listed in random order (see survey, https://osf.io/7q6zh [accessed on 3 November 2023]). In Table 2, however, jobs are grouped into “job clusters” that were derived from analyses of the JSR survey data, as explained in detail in the first subsection of the results.

#### 2.2.3. Measures Administered to the SCIBS Sample

Participants in the SCIBS sample were given four measures, described below. These four measures were always presented in the same order (self-concepts, job interests, spatial task, **and** job stereotyping beliefs). On all but the spatial task, the orders of items were varied within a measure across participants. A sample survey using one of the random orders is posted here (https://osf.io/fjt6m/ [accessed on 3 November 2023]). 

**Self-Concept Ratings in Six Domains.** We asked participants to report on their self-concepts in each of six domains. In addition to asking about our key focus—spatial skills—we asked about two domains commonly associated with STEM occupations—math and science. As a fourth domain, we added English, selected because this skill is emphasized in K-16 curricula but is not routinely associated with STEM. Finally, we included two other skill domains—foreign language and athletics—to provide additional non-STEM comparisons.

For each domain, we gave participants 10 statements and asked them to select which of seven levels of agreement conveyed how true each statement was for them. Response options ranged from *completely false* (scored as 1) through *neutral* (scored as 4) to *completely true* (scored as 7), so that higher scores represent stronger personal endorsement. Drawing on work by Eccles and colleagues (e.g., Denissen et al. 2007), we included among the 10 statements three subtypes of items: skill competence (six items; e.g., “I have always done well in science”), skill value (two items; e.g., “In general, I think that developing my science skills is useful to me”), and skill liking or interest (two items; e.g., “I really like working on science a lot”).

Results showed that participants’ responses to the statements about competence, value, and liking were highly correlated within domains. Thus, responses within each domain were averaged to create an overall self-concept score, with the proviso that no more than one item in a single domain had been skipped. Very few participants skipped more than one item within a given domain, and so no more than two participants were missing a score in any one domain. Internal consistency reliabilities measured via coefficient alpha were >.90 for all six domain scales.

**Interests in Specific Jobs.** Participants were asked to rate their personal interest in each of the 82 specific jobs used in the JSR survey using the following response options: *not at all; a little; some; pretty much;* or *very much*. Responses were scored from 1 to 5; thus, higher scores represented greater interest. Analyses of data from the JSR sample were used to form subscales for this measure as described in the Results.

**Beliefs about Job Cultural Gender Stereotypes.** Participants were asked to report their beliefs about “whether people in our culture generally think about these jobs as for men, for women, or for both men and women”. Participants were asked to “indicate what you think most people believe about each job” by choosing one of five responses: *Only for men; Mostly for men and a few women; Equally for men and women; Mostly for women and a few men;* and *Only for women*. Responses were scored from 1 to 5. Thus, mean scores around the midpoint (3) indicate jobs viewed as culturally gender neutral; those below 3 indicate jobs viewed as culturally masculine; and those above 3 indicate jobs viewed as culturally feminine. In parallel to the job interest measure and, again, as described in more detail in the Results, data from the JSR sample were used to form subscales for this measure of beliefs about cultural job stereotypes.

**Spatial Performance.** Participants were given a water level task (WLT) as a measure of their spatial skill. The WLT was designed by Piaget and Inhelder (1956) to assess children’s developing construction and use of systematic spatial representational systems (such as a Cartesian coordinate system of horizontal and vertical axes) to perceive and represent abstract or observable phenomena (such as the horizontal position of the water surface in a half-filled, tilted bottle). Later research demonstrated that many adults—particularly women—also have difficulty on the WLT, and that individual differences in the WLT are correlated with performance on other spatial measures and with STEM performance (e.g., Liben et al. 2011). We selected the WLT as the spatial measure because of its practical advantages: it is short and untimed, and unlike many spatial assessments, is typically enjoyed by children and adults, irrespective of how well they perform on the task.

The WLT used here contained 15 drawings of containers of varying shapes oriented at various angles, each with an embedded water line. In a third of the items, the line was drawn correctly so that the line was parallel to the table and the bottom of the computer screen. In the remaining items, the water line was drawn incorrectly, deviating from horizontal between 8° and 15°. For each item, participants were asked, “Does the line show where the water would be if the container is held in this position?” and asked to select either “Yes” or “No”. Performance was scored as the total number correct. Internal consistency reliability measured via coefficient alpha was .89.

#### 2.2.4. Data Collection

The initial wave of data collection using the JSR survey (*n* = 65) and the SCIBS survey (*n* = 149) took place in February 2011; some data from these 214 participants were reported as part of an undergraduate honors thesis (Angle 2011) that focused on the SCIBS survey questions and on perceptions of 11 obscure or novel jobs used in earlier research (Liben et al. 2001). Additional data for the current study were collected later in the same semester (April 2011) to reach the planned sample sizes of approximately 300 participants per survey. The second recruitment phase provided an additional 235 participants for the JSR survey and 143 for the SCIBS survey. The JSR and SCIBS surveys used in February and April were identical, with the exception that in April, questions about the 11 obscure or novel jobs were omitted.

## 3. Results and Discussion

We begin by reporting and discussing results from the first research question using the JSR survey responses. We then explain how we used the JSR results to structure our analyses of results from the SCIBS survey to address the second research question.

### 3.1. Research Question 1: Can Students Identify Spatial and Other Skills Needed for Specific Jobs?

#### 3.1.1. Cluster Analysis

Cluster analysis of the JSR data was used to identify job skill groupings based on the skill requirements for the various jobs and to test whether ratings for spatial skills contributed to student-derived job groupings. If students associate spatial and other skills consistently with specific jobs, then these job skill groupings would emerge in the cluster analysis. Clusters identified in this way could then be cross-validated with data from O*NET. Table 2 shows the derived clusters and relations to O*NET data, described in detail below.

The data used for the cluster analysis were the average ratings across participants for each of the 82 occupations on math, spatial, English, and science skills. Given that participants were told that they could skip items as needed (e.g., for not knowing a job), there were occasional (but rare) missing data. Of the 300 participants in the sample, the smallest number of responses contributing to a mean rating was *n* = 270, and half or more of the items elicited responses from 298 to 300 participants.

The mean ratings for the 82 jobs on the four skill dimensions were first subjected to a hierarchical cluster analysis (SPSS 29), with no *a priori* specification of the number of clusters. The resulting dendrogram showed a two-cluster solution that created very large heterogenous clusters, whereas the six-cluster solution had clusters with very few items in them. We judged that the four-cluster solution provided the best balance. The second step was a cluster analysis using the k-means solution to test the four-cluster configuration further. The solution converged after three iterations. The descriptive ANOVAs supported the conclusion of distinctiveness among the clusters (all *p*s < .001).

Table 2 shows the four skill ratings for each job, grouped by the cluster structure derived from the k-means analysis. We named the clusters based on which of the four skills received the highest average ratings (maximum rating = 5) and our judgment about the nature of the jobs included within the cluster. The ***quantitative*** cluster (11 jobs) contained occupations involving math, statistics, finance, or accounting. In this job cluster, math skills elicited the highest average ratings (*M* = 4.40), followed by English (*M* = 3.05) and spatial skills (*M* = 3.05), and then science skills (*M* = 2.64). The ***verbal*** cluster (six jobs) was characterized by higher skill ratings for English (*M* = 4.25) than for any other skill. Jobs in this cluster rely heavily on spoken and/or written language (e.g., writer and comedian). The ***basic & applied science*** cluster (38 jobs) included occupations in the physical, biological, and social sciences; engineering; and medical professions. In this cluster, the highest ratings were assigned to science skills (*M* = 4.41), followed by math (*M* = 4.02), spatial (*M* = 3.72), and English skills (*M* = 3.08). The ***spatial*** cluster (27 jobs) contained jobs that received relatively high spatial skill ratings (*M* = 3.44). Jobs in this cluster included diverse occupations, some were technical (e.g., auto mechanic) and some were artistic (e.g., ballet dancer). Few of these jobs require advanced degrees. In the spatial cluster, participants’ ratings in the other three domains (English, science, and math) were similar to one another and lower than the spatial rating.

To test further the structure of the clusters and whether they differed by participant gender, we conducted a mixed models ANOVA in which the factors were participant gender (woman or man), job skill cluster (quantitative, verbal, basic & applied science, and spatial), and skill rating (math, English, science, and spatial), with repeated measures on job skill cluster and skill rating. Because of the method used to form the clusters, an interaction between the job skill cluster and skill rating was anticipated and obtained, *F*(9, 4076) = 729.7, *p* < .001. Follow-up tests with Bonferroni corrections showed significant differences in the skill patterns within the clusters described in the previous paragraph.

The overall ratings by women (*M* = 3.20) and men (*M* = 3.11) were not significantly different, *F*(1, 281) = 2.71, *p* = .10. Most crucially, there was no significant interaction of participant gender with the job skill cluster and skill rating, *F*(9, 4076) = 1.34, *p* = .21, thus providing no evidence that cluster patterns differed significantly by participant gender.

#### 3.1.2. Linking JSR Skill Clusters to O*NET STEM Designations and Skill Ratings

Skill ratings by O*NET job analysis professionals provide a method to cross-validate the student ratings from the JSR survey and the job skill clusters that were obtained from those ratings. Table 2 includes a column labeled O*NET with two sub-columns summarizing available O*NET data for jobs included on our list. The complete compilation of our jobs and their links to O*NET data is available at https://osf.io/6dby2/ (accessed on 3 November 2023). Missing information occurred for several reasons. In some cases, O*NET had combined jobs we had listed separately (e.g., farmer and rancher named separately on our surveys were grouped together under “farmers, ranchers, and other agricultural managers” on O*NET). In other cases, O*NET had incomplete data for a job on our list (e.g., financial analyst). In a few cases, we found no viable counterpart on O*NET for a survey-listed job (e.g., supermarket owner).

The O*NET STEM column of Table 2 shows whether each job on the JSR survey was classified as a “STEM” job on O*NET (NCOD 2023e). Recall that the O*NET STEM designation refers to jobs requiring science, technology, engineering, and math education. The ratings from the two sources (i.e., JSR participant ratings and O*NET designations) were largely consistent. Most notably, 91% (30/33) of the basic & applied science cluster jobs that were also listed on O*NET were included in the O*NET STEM category. The student ratings, which grouped jobs that called on, in order, science and math, were therefore consistent with O*NET’s category and definition.

The next closest match between JSR and O*NET STEM ratings was evident in the quantitative cluster: 44% (4/9) of the jobs in the JSR-derived quantitative cluster that were also included on O*NET were listed as STEM jobs on O*NET. The ratings by JSR participants showed math as the dominant skill, with lower averages for the other three skills. By O*NET’s own definition of STEM, we can infer that math-dominated jobs that do not also have another significant component such as science would, therefore, not be categorized as STEM. The four jobs in the JSR quantitative category that *were* viewed as STEM by O*NET had the highest science ratings in that group (actuary, mathematician, statistician, and systems analyst). Again, the student raters’ perceptions aligned with the judgments of the professional job analysts.

None of the jobs categorized into the verbal cluster via the JSR data was categorized as a STEM job on O*NET.

Of the 25 nonduplicate jobs from our spatial cluster that were also on O*NET, only two were included on O*NET’s STEM list (8%; 2/25). We do not take this observation to imply that either our raters or O*NET analysts consider spatial skills unimportant for STEM jobs. Instead, we interpret this finding to be a consequence of our decision to include jobs from non-STEM domains on our list of “spatial” jobs (e.g., jobs from the visual or performing arts).

To address more directly the question of the judged importance of spatial skills for STEM jobs or any other occupations, we next examine the importance ratings for visualization given by O*NET analysts (NCOD 2023a) to the jobs in our survey. Recall that O*NET includes two sub-abilities under its category of spatial ability. One of these—visualization—is defined on O*NET as “the ability to imagine how something will look after it is moved around or when its parts are moved or rearranged” (NCOD 2023c). As defined by O*NET, visualization seems a more general skill and a better fit to frequently used paper-and-pencil spatial assessments. Visualization was more frequently rated as important for the jobs listed on the JSR survey than was the other sub-ability—spatial orientation—defined on O*NET as “the ability to know your location in relation to the environment or to know where other objects are in relation to you” (NCOD 2023b). Among the 70 unique jobs on the JSR survey for which there are O*NET abilities data, only three (airplane pilot, ship captain, and truck driver) had a spatial orientation ranking > 3, thus contributing to our decision to focus on visualization rather than orientation ratings on O*NET.

The O*NET Spatial Visualization column of Table 2 shows the ratings of importance given to each job by the O*NET analysts. The scale ranges from 1–5, with a rating of 1 indicating that the ability is judged as not relevant.

Two descriptive comparisons show correspondence between student ratings and the O*NET analysts’ ratings of the importance of spatial visualization for jobs. First, a one-way ANOVA comparing the O*NET spatial visualization rating by job cluster was significant, *F*(3, 66) = 8.49, *p* < .001. Follow-up tests with Bonferroni corrections showed that the spatial visualization ratings for the basic & applied science cluster (*M* = 3.1) and spatial cluster (*M* = 3.0) were significantly higher than for the quantitative cluster (*M* = 2.5) and verbal cluster (*M* = 2.3). Across all clusters, the students’ JSR spatial skill ratings were highly correlated with the O*NET analysts’ ratings for spatial visualization, *r*(68) = .75, *p* < .001.

The patterns of data from participants’ ratings are especially interesting insofar as they offer some evidence that college students’ everyday beliefs about which foundational skills are needed for various jobs are similar to conclusions about foundational job skills identified by career counselors, educators, and vocational and occupational research experts; derived from clinical experience; and arrived at by various analyses of large data sets that track occupational choice and success over time and in relation to measured skill sets (e.g., Wai et al. 2009; Le et al. 2014). Student ratings in our research yielded a “basic & applied science” job cluster that aligns with typical definitions of STEM and includes an important spatial component, although we note that in this cluster, spatial skills were judged to be less important than were general science skills and math skills. The student ratings also produced a “spatial” job cluster in which spatial skills were the most important skills in the cluster and aligned with job analysts’ ratings of the importance of visualization, but which did not have the science and math skill components that are typically associated with a STEM designation. The data in hand, therefore, provide evidence that spatial skills are salient in students’ understanding of job requirements.

In the next section, we examine the SCIBS survey data, focusing on whether students’ own self-concepts, beliefs, and spatial task performance are related to their interests in STEM or other jobs.

### 3.2. Research Question 2: Do the Job Skill Groupings Increase Understanding of Emerging Adults’ Reported Interests in Pursuing STEM Careers?

Our goal in analyzing the SCIBS survey data was to test whether the job skill clusters were related to previously identified factors from research and models examining achievement and interest pathways (Eccles and Wigfield 2020; Martin et al. 2002). Specifically, we focused on factors previously identified as potential deterrents to women entering STEM fields. First, we present descriptive information on the SCIBS survey measures, including the scales based on the job skills clusters. Second, we use the resulting scales to predict the students’ reported job interests.

#### 3.2.1. Descriptive Information

Table 3 presents the means, standard deviations, and effect sizes for the four measures administered to the SCIBS sample, reported separately by participant gender. These measures include those tapping (1) self-concepts in specific skill domains, (2) job interests, (3) beliefs about cultural job gender stereotypes, and (4) spatial performance. The four job clusters identified from the JSR survey (i.e., quantitative, verbal, basic and applied science, and spatial) were used to create subscales for examining job interests and stereotype beliefs.

Before turning to more detailed findings, we note that most—but not all—measures had internal consistency reliabilities that were acceptable or better. Specifically, reliabilities for three of the job interest subscales were strong (alphas for these subscales were as follows: quantitative = .91; basic & applied science = .94; and spatial = .88), but one was not (verbal = .58). For the cultural gender stereotyping subscales, there were problems on the verbal subscale insofar as alpha was near zero due to a lack of coherence among the items, and on the spatial subscale insofar as alpha was unacceptably low (.52). These issues are also noted in Table 2 (in the footnotes) and in the descriptions of the analyses. As reported earlier in the Methods, reliabilities for the self-concept skill domains and for the water-level task were good.

**Self-Concepts by Domain.** A MANOVA of the self-concept measures by participant gender was significant using Pillai’s trace, *V* = 0.12, *F*(6, 279) = 6.23, *p* < .001. Critical values for the univariate tests were adjusted to *p* < .0083. Women’s self-concept scores were significantly higher than men’s for English (*p* < .001) and foreign language (*p* = .003), whereas men’s were significantly higher than women’s for athletics (*p* = .001) and math (*p* = .003). Men’s averages were higher than women’s for science and spatial skills, but these differences were not statistically significant. Adjusting the *p*-value to be more conservative affected only the conclusion about the significance of the gender difference in science self-concept. In the spatial self-concept, no significant gender difference would be present even if the probability threshold were set more leniently at *p* < .05. The directions of the gender differences detailed above and in Table 3 were generally consistent with prior research (e.g., Jiang et al. 2020). Still, we note that we did not find as large a self-concept advantage for men in spatial skills as has been reported by other researchers (e.g., see Matthews et al. 2024, p. 100).

**Job Interests by JSR Clusters.** Subscales composed of the average job interest scores for the four job clusters identified from the JSR survey (quantitative, verbal, basic & applied science, and spatial) were the dependent variables in a MANOVA with participant gender the between-subjects factor. Using Pillai’s trace, there was a significant effect of participant gender, *V* = 0.16, *F*(4, 278) = 12.79, *p* < .001. Three of the four subscales had significant gender effects using the critical value of *p* < .0125. Men had significantly higher average interest than women in the quantitative cluster jobs (*p* = .002) and in the basic & applied science cluster jobs (*p* = .01), whereas women had significantly higher average interest than men in the spatial cluster jobs (*p* = .011). These three subscales were also the ones that had good internal consistency reliabilities. The two job clusters in which men had greater interest than women (basic & applied science and quantitative) were also the clusters with the greatest numbers of jobs considered STEM jobs by O*NET (see Table 2). The spatial cluster has very few jobs considered STEM by O*NET.

**Gender Stereotyping of Jobs by JSR Clusters.** As with job interests, subscales composed of the average stereotyping scores for the four job clusters derived from the JSR survey (quantitative, verbal, basic & applied science, and spatial) were the dependent variables in a MANOVA with participant gender as the between-subjects factor. Using Pillai’s trace, there was no significant gender difference in the scores, *V* = 0.03, *F*(4, 283) = 2.16, *p* = .07.

To test whether the average stereotyping scores deviated significantly from the midpoint of 3 (the response option for judging jobs as “Equally for men and women”), *t*-tests were performed on each subscale, with a two-tailed critical value of *p* < .0125. In all four clusters, the means deviated significantly from the midpoint of 3. The quantitative cluster (*p* < .001, *d* = 0.48), basic & applied science cluster (*p* < .001, *d* = 0.35), and spatial cluster (*p* < .001, *d* = 0.21) all averaged below 3 and therefore were tipped in the stereotypically masculine direction. The verbal cluster averaged above 3 (*p* < .001, *d* = 0.26) and therefore was tipped in the feminine direction. Thus, the jobs that are considered to be STEM and/or to have significant math or spatial components were believed by the participants to be stereotyped in the culture as masculine, consistent with recent research (Master et al. 2021).

**Spatial Performance on the Water-Level Task.** Men’s WLT scores were significantly higher than women’s, *t*(291) = −3.25, *p* = .001, *d* = −0.38, a gender difference consistent with prior work (e.g., Vasta and Liben 1996).

#### 3.2.2. Structural Equation Models Predicting Job Interest

We used structural equation modeling to test whether STEM-related job interests are predicted by the other measures on the SCIBS survey. In our initial grant proposal and early public presentations of our research (e.g., Liben et al. 2017), our models were largely motivated by the prior work of Eccles and colleagues that also informed our choice of self-concept measures (e.g., Denissen et al. 2007). In our current work, we have drawn from the updated SEVT theory by Eccles and colleagues (Eccles and Wigfield 2020). This newer version elaborates on factors such as cultural stereotyping and previous achievement as impacting one’s academic self-concept and achievement-related choices. SEVT also includes potential indirect pathways from stereotyping and previous achievements to self-concept and eventual achievement-related choices. Structural equation modeling allows us to test these direct and indirect effects.

Several additional considerations led to the pathways we tested based on SEVT. First, we omitted any measures that lacked adequate reliability. Second, patterns that emerged from the analyses of the JSR sample suggested that student raters (and O*NET analysts) did not view the spatial job cluster as STEM. Thus, we focused on job interest outcomes from the two job clusters with the strongest STEM associations—basic & applied science and quantitative. Finally, to represent the spatial components relevant to STEM, we used spatial self-concept and water-level task performance. We did not use spatial job cluster gender stereotyping because it lacked adequate reliability, likely a consequence of our having included a wide assortment of spatial jobs from disparate domains, as discussed earlier. The intercorrelations for all measures for the sample as a whole and separately by gender are shown in Tables S1 and S2 in the Supplemental Materials (https://osf.io/874ew/ [accessed on 3 November 2023]). 

The models for the two STEM outcomes—interests in basic & applied science jobs and interests in quantitative jobs—were analyzed using SPSS AMOS (see also, Collier 2020). For each outcome, two-group analyses comparing women and men were conducted to test for gender differences. When gender differences were significant, additional tests were conducted to locate the source(s) of those differences.

**Figure 1:**
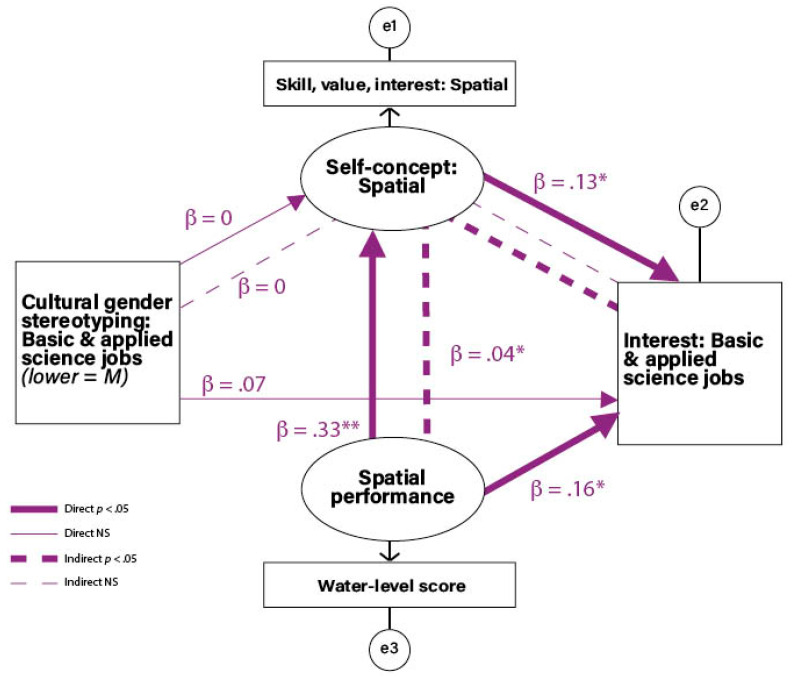
Structural equation model predicting interest in basic & applied science cluster jobs. (* *p* < .05; ** *p* < .01).

**Predicting Interest in Basic & Applied Science Jobs.** The results from testing the model predicting interests in the basic **&** applied science jobs are shown in Figure 1. The predictors were cultural gender stereotyping of basic & applied science jobs (hereafter abbreviated in the text as *science stereotyping*), spatial self-concept, and spatial performance. The two latent factors were spatial self-concept, represented by the spatial skill, value, and interest indicator, and spatial performance, represented by the score on the water-level task.

The prediction based on SEVT was that there would be three direct effects. Two of these were that science stereotyping and spatial performance would each predict spatial self-concept. The third expected direct effect was that spatial self-concept would predict basic & applied science job interest. Also expected from the model were indirect effects of science stereotyping and of spatial performance on job interest, mediated through spatial self-concept. For completeness, the model included a direct link from spatial performance to job interest to allow for the possibility that performance on a spatial task might impact job interest independent of spatial self-concept. Similarly, the direct effect of science stereotyping on job interest was tested, as this path is implicit in many gender schema models and theories (e.g., Martin et al. 2002).

In the test of the model, the first step was a two-group analysis by gender, which did not show significant gender differences. The best-fitting model had equal path values for women and men, χ^2^(11) = 13.783, *p* = .245, CFI = .929, RMSEA = .030. Therefore, the genders were combined and the coefficients in Figure 1 and Table 4 are for the combined sample, χ^2^ (1) = 0.263, *p* = .608, CFI = 1, RMSEA = 0. As predicted, there was an indirect effect of spatial performance on basic & applied science job interest mediated through spatial self-concept. Additionally, as predicted, there were direct effects of spatial performance on spatial self-concept, and of spatial self-concept on basic & applied science job interest. Contrary to predictions, science stereotyping was not associated with any other variables.

This model suggests that women’s and men’s interest in basic & applied science jobs—the jobs most likely to be viewed by O*NET analysts as STEM—are at least partially related to an aspect of spatial skill and that the relation is both direct and indirect via spatial self-concept. These data are, of course, correlational, and so the impact may be in either direction or bi-directional. Nonetheless, the findings do demonstrate connections among spatial performance, self-concept, and interest in basic & applied science cluster jobs. A question for future research is whether greater attention to spatial skills in K-12 curricula and in career exploration programs can promote stronger spatial skills and spatial self-concepts, particularly among those who have not traditionally excelled in those areas. An affirmative answer to this question would provide support for implementing a recommendation made by the National Research Council (2006) to infuse spatial skill education into substantive instruction across multiple disciplines. In the long term, this approach may be expected to attract more—and more diverse—students to become interested in, pursue, and succeed in STEM jobs.

The failure to find a connection between science stereotyping and any of the other variables may be due to one or both of the following factors. First, although the overall rating of the basic & applied science cluster jobs was in the masculine direction, there was variation in gender stereotype ratings for individual jobs within this cluster. Many traditionally masculine jobs retained their traditional masculine stereotype (e.g., engineer and computer scientist), but others that may have previously been considered masculine were rated as slightly feminine (e.g., psychologist and veterinarian). Also included in the basic & applied science cluster were historically feminine jobs in science-related fields that remained so (e.g., nurse and physical therapist). Second, the form of the stereotyping measure we used was one in which participants were asked to report on what “most people believe” rather than to report on what they *themselves* believe about each job. The former type of question has been said to tap respondents’ knowledge of cultural gender stereotyping whereas the latter (e.g., “who *should* perform [named job]?”) has been said to tap respondents’ *own* attitudes or stereotype endorsements (Liben and Bigler 2002; Signorella and Liben 1985). Consistent with this distinction are meta-analyses showing that different questions yield different response patterns (Signorella et al. 1993). Both aspects of gender stereotyping need to be studied. For example, in the SEVT model (Eccles and Wigfield 2020), an individual’s own perceptions of stereotypes mediate the relation of cultural stereotypes and self-concepts. We assessed the knowledge of the culture’s gender stereotypes in the present research because there was little empirical work on how the spatial component in particular was viewed, but it would also be important for future investigators to assess individuals’ personal acceptance of gender stereotypes of jobs that have significant spatial components.

**Predicting Interest in Quantitative Jobs.** Next, we consider participants’ reported interests in the quantitative job cluster. The initial two-group analysis tested a model in which there were no gender differences. The no gender difference model, however, was a poor fit, χ^2^ (6) = 24.781, *p* < .001, CFI = .619, RMSEA = .107. To achieve a model that fit the data, constraints were relaxed to allow for differences between women and men.

**Figure 2:**
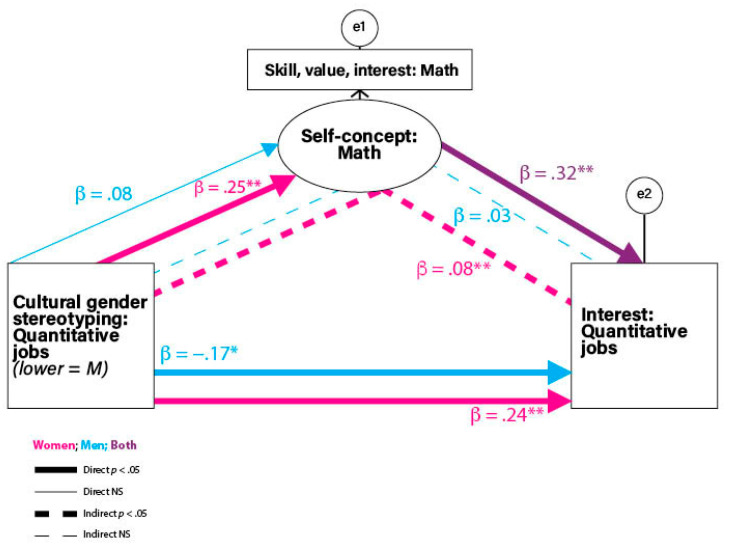
Structural equation model predicting interest in quantitative cluster jobs. (* *p* < .05; ** *p* < .01).

The best-fitting model was produced by removing the no difference constraints on two paths: cultural gender stereotyping of quantitative jobs to math self-concept and cultural gender stereotyping of quantitative jobs to quantitative job interest, χ^2^ (3) = 3.634, *p* = .304, CFI = .987, RMSEA = .028. Figure 2 shows the standardized path coefficients for women and men; Table 5 shows the tests of the direct and indirect effects. Among women, less stereotyping of quantitative cluster jobs as masculine (i.e., higher scores) was directly related to women’s greater interest in the quantitative jobs and was related indirectly through the math self-concept. Thus, the analyses show a partially mediated pattern for women. In contrast, among men, there was a direct association between more masculine stereotyping of jobs in the quantitative cluster (i.e., lower scores) and greater interest in those jobs. Among both women and men, there was a direct association between a higher math self-concept and greater interest in quantitative jobs. Thus, among men, there was no evidence of mediation through self-concept from stereotyping.

The pattern for women, therefore, was one in which the cultural stereotyping of quantitative jobs as masculine was both directly and indirectly related to interest in those jobs. Even though performance in math in the United States is now comparable across genders (Hyde 2014), our data showed that compared to men, women had less interest in the jobs in the quantitative cluster. The finding that lower stereotyping of quantitative jobs as masculine was associated with women’s greater interest in quantitative jobs contrasts with the absence of parallel findings from the model examining predictors of women’s interest in basic & applied science jobs. Our data do not allow us to determine the reason for the different patterns in the two job clusters. Perhaps the cultural stereotyping of math as masculine (e.g., Nosek et al. 2002) is stronger than the parallel cultural stereotyping of science, or perhaps the differences in data patterns might be traced to different levels of job diversity within each of the two job clusters. Additional studies with larger and more diverse samples of both participants and jobs are needed. In the final section below, we highlight key findings, as well as the limitations of the current work and offer some suggestions for next steps.

## 4. Conclusions, Cautionary Notes, and Implications for Future Research

The results from students’ ratings of skills needed for specific jobs (data from the JSR survey) suggest that college students have reasonably sophisticated ideas about what is needed for specific occupations. Our participants’ assessments of what skills are needed for STEM careers were similar to the assessments provided by O*NET professionals. Our participants also identified jobs that required spatial skills that were not included by O*NET in the STEM category, but which O*NET analysts also identified as needing spatial skills. We found no evidence that women and men differed in how they associated skills to jobs. Thus, by the time they reach college, both women and men have some understanding of spatial skills and recognize their relevance for various occupations. Interventions to increase interest in STEM careers may thus not need to begin by convincing students that spatial skills are important for STEM. Such beliefs are already present.

The results from the structural equation models provide evidence of connections between performance on a spatial task and other outcomes. With respect to the outcome of participants’ interest in basic & applied science jobs, the analysis showed no differences between patterns in women and men. Specifically, irrespective of self-reported gender, the WLT score was directly related to the spatial self-concept and basic & applied science job interest, and indirectly related to basic & applied science job interest through the spatial self-concept. Better performance on the WLT and other Piagetian spatial measures has been linked to children’s and adults’ successes in acquiring STEM-related concepts and skills (e.g., Bower and Liben 2021; Liben et al. 2011, 2013).

The model predicting interest in the quantitative cluster jobs showed both similarities and differences between women and men. In both gender groups, the math self-concept was directly related to interest in the quantitative cluster jobs. However, in women (but not in men), *less masculine* stereotyping of quantitative cluster jobs (as indicated by *higher* numbers on the cultural job stereotyping measure) was directly associated with a higher math self-concept and with greater interest in the quantitative cluster jobs. In contrast, *more masculine* stereotyping of quantitative cluster jobs by men (as indicated by *lower* scores on the cultural job stereotyping measure) was related to higher interest in those jobs. The other difference in the patterns by gender was that the relation between women’s lower masculine stereotyping of the quantitative cluster jobs and women’s greater interest in those jobs was mediated by the math self-concept.

Taken together, these findings suggest that fruitful avenues for reducing gender disparities in STEM careers include facilitating the development of spatial skills by all students and reducing the persistent perception that mathematics and other quantitative jobs are stereotyped as masculine in the culture. These suggestions are not intended to ignore the possibility that interests in math and quantitative jobs reduce women’s tendency to stereotype these careers as masculine, nor are they meant to rule out the possibility that effects are occurring in both directions. In addition, these suggestions are not intended to rule out or ignore contributions of other factors that may deter women from STEM. For example, interventions are also needed to change gender-differentiated family expectations that limit girls’ participation in STEM-relevant activities, ensure that educators provide activities and curricula that encourage girls’ interest in STEM careers, and redress hostile STEM environments that lead girls and women to avoid or withdraw from STEM fields (e.g., Kitchen et al. 2023; Wang and Degol 2017).

### 4.1. Limitations

The biggest limitations to the results from the current work lie in two areas. One limitation is that—even in the face of finding considerable agreement about necessary job skills between our college student participants and O*NET’s professional analysts—we have no direct evidence about whether students’ beliefs about job-related skills do, in fact, impact students’ actual choices about majors and careers (not simply their responses to survey items). Other researchers of achievement motivation and career development (e.g., developers of SCCT and SEVT) have provided evidence similar to the evidence we provided based on our model analyses of SCIBS data. Still missing from both prior and current work are demonstrations that the pathways from spatial performance to spatial self-concept are precursors to individuals’ real-life career choices and successes.

The second limitation, noted earlier, concerns the substantial variation in judged cultural gender stereotyping of the jobs falling into the basic & applied science job cluster. This variability may be responsible for the absence of associations between basic & applied cultural job stereotyping and any of the other factors. Thus, even though, on average, jobs in the basic & applied science cluster are rated as culturally stereotyped in the masculine direction, the inclusion—within the same cluster— of jobs perceived as neutral or feminine may attenuate associations between the cluster of jobs as a whole and the associations with other factors we examined.

Related to the second limitation is the observation that there were also considerable variations in the jobs that fell into each of the job clusters we identified via the JSR rating data. Our sample sizes (of both participants and jobs) do not allow us to identify and examine all potential subsets of jobs within each cluster. For example, within both the verbal and spatial clusters are some jobs that are usually found to be stereotypically masculine and some that are usually found to be stereotypically feminine or neutral. The spatial job cluster derived from our 82 jobs also includes a disproportionately large number that do not require higher education, and the definition of spatial skills we used was more general than that employed by O*NET. Within the basic & applied science cluster, there are variations in how crucial spatial skills would be, based on the O*NET job analyst data. In future examinations of the spatial skill components of jobs, these additional factors should be explored more fully and systematically.

Given that the survey responses we studied in this research were collected in 2011, a reasonable question is whether there are likely to have been any dramatic changes during the last decade. As reviewed in the Introduction, women have made progress in reducing or even closing academic major and employment gaps in some STEM areas (e.g., biology) but still lag behind men in others (e.g., computer science and engineering). With respect to gender stereotyping, the question is not whether there have been changes across the last century or so, which are well-documented (e.g., Liben 2016), but rather whether there have been noticeable shifts more recently. A comparison by Haines et al. (2016) between 1983 and 2014 is particularly relevant. They asked participants “to estimate the likelihood that a man, a woman, or a person with gender unspecified had a set of male-typed and female-typed characteristics” (p. 356). Gender stereotyping of traits, occupations, and masculine role behaviors did not change. The only significant change was in the direction of greater stereotyping of feminine role behaviors.

In slightly later work, Eagly et al. (2020) conducted a meta-analysis of public opinion polling data on gender-related traits. They reached a different conclusion, arguing that the results showed change in gender stereotypes. There is agreement between the two studies insofar as both show increases in stereotyping over time in aspects of feminine stereotypes (role behaviors, Haines et al. 2016; communal traits, Eagly et al. 2020). There is also some agreement that areas of masculine stereotypes have not changed (masculine traits and roles, Haines et al. 2016; agentic traits, Eagly et al. 2020). The change identified by Eagly and colleagues was in the general characteristic of competence and specific items about intelligence, which were shown to have become more neutral and less strongly stereotyped as masculine. This change may seem inconsistent with the recent data from research with children showing a masculine stereotype of intelligence (e.g., Bian et al. 2017, 2018), but the Bian studies are examining high intelligence or brilliance, whereas the items used in the surveys summarized by Eagly et al. were not. Specifically, the traits Eagly et al. used included logical, common sense, and organized, and none included brilliant or similar descriptions. An important conclusion suggested by Eagly et al. (2020) that also provides directions for future research was that increased communal stereotyping of women, combined with continued masculine stereotyping of agency and increased attribution of competence or intelligence to women, might explain the increases in women choosing the less masculine specialties found in traditionally masculine fields (e.g., pediatrics).

### 4.2. Important Future Considerations

Researchers who study STEM gender stereotyping, self-concept, and job choice also need to consider possible intersectional variations in these stereotypes and their impacts on choices of majors and careers (Signorella 2020). Recent NSF data show that “Women, persons with disabilities, and persons from some racial and ethnic minority groups—Hispanic or Latino, Black or African American, and American Indian or Alaska Native—are underrepresented in the STEM workforce when compared to their share of the total population” (NCSES 2023, p. 5). All disparities, not just gender, require attention; likewise, intersections of identities and experiences (e.g., those associated with gender, SES, race, and ethnicity) must be addressed. Given that we drew from a population that was not diverse, was predominantly White, and for which we had no information on many important demographic and self-identified variables that might well affect actual or imagined career possibilities (e.g., immigrant history, SES, or disability), our findings cannot be assumed to generalize to all people and populations. There is some research suggesting that there are differing and complex pathways from self-concepts to career achievement by gender and race that are resulting in the gender and racial disparities seen in STEM career outcomes (Seo et al. 2019).

### 4.3. Conclusions

There is considerable evidence that girls and women tend to score lower than boys and men on many spatial measures. In the present research, we found that college women and men—much like professional job analysts and counselors—perceive spatial skills to be relevant for many jobs. We also found that spatial performance was related (both directly and indirectly through spatial self-concept) to interest in basic and applied science jobs, the category most like O*NET’s definition of STEM. Thus, our research identifies possible real-world impacts of spatial skills on students’ interest in STEM careers, both directly and indirectly through spatial self-concept.

We acknowledge that the research we report in this paper leaves many important questions unanswered. At the same time, we suggest that the data in hand offer empirical support for previously under-tested assumptions or hypotheses. Our data support the general proposition that students harbor beliefs about the relevance of spatial skills for various careers and that these students’ beliefs are well-aligned with beliefs of career analysts. Data also demonstrate that students have beliefs about their own level of spatial skill competence and about the ways that the surrounding culture views particular science-related job clusters as gendered. Importantly, students’ reports of their own skills and beliefs about societal attitudes are connected to their own job interests. Taken together, our findings suggest that intervening before college to improve students’ spatial skills is a reasonable recommendation. Future work will need to address whether individuals’ actual choices of majors and careers follow patterns like those found in students’ responses to survey questions about job interests.

## Figures and Tables

**Table 1:** Format for the Job Skills Rating (JSR) Survey: Instructions and illustrative job.

**General Instructions**					
Thinking about Jobs					
This survey asks you questions about jobs. We are trying to learn what skills people think different jobs require. We will ask you to judge about 100 jobs by rating how much each one calls upon English, math, science and spatial skills.					
For MATH, try to think about math skills in general rather than about one specific type of math; think of the general ability to work with numbers, perform calculations, and solve mathematical problems.					
For SPATIAL SKILLS, try to think about spatial skills in general rather than about any one particular skill; think of the general ability to visualize or mentally manipulate shapes, patterns, or spaces.					
For ENGLISH, try to think about English skills in general rather than about any one particular skill, such as vocabulary or spelling; think about English skills as the general ability to effectively communicate in the English language.					
For SCIENCE, try to think about science skills in general rather than any one scientific discipline; think about science as the general ability to understand and apply the scientific method.					
You will be answering each question using a 5-point scale as follows: the job calls on [NAMED SKILL] 1 = not at all 2 = a little bit 3 = some 4 = pretty much 5 = a lot					
We generally find that the 82 jobs we list are familiar to most people, and thus we have not included any job descriptions. If you don’t know a job at all, just skip that job.					
We know that there are a lot of jobs, but would ask you to try to think about each one carefully when you answer. Despite the number of questions, they are short, and thus you should be able to finish within 30 min.					
Illustrative job showing how each job appeared on the survey					
**1. LIBRARIAN calls on**					
	not at all	a little bit	some	pretty much	a lot
Math	O	O	O	O	O
Spatial skills	O	O	O	O	O
English	O	O	O	O	O
Science	O	O	O	O	O

**Table 2:** Cluster analysis of job clusters: Average ratings by skills for each job and each cluster and relations to O*NET data.

		Average Ratings by Skill				O*NET	
Quantitative Cluster	Distance from Cluster Center	Math	English	Science	Spatial	STEM ^a^	Spatial Visualization ^b^
Financial Analyst	0.41	4.67	3.03	2.65	2.75	N	n/a
Company Treasurer	0.45	4.60	3.22	2.30	2.91	N	2.38
Financial Clerk	0.47	4.56	3.07	2.33	2.74	N	2.00
Accountant	0.58	4.83	3.08	2.26	2.95	N	2.25
Actuary	0.59	4.01	2.87	3.02	2.91	Y	2.50
Statistician	0.67	4.84	2.79	3.08	3.03	Y	2.75
Loan Officer	0.88	4.45	3.47	1.91	2.79	N	1.88
Mathematician	0.89	4.87	2.40	3.02	3.18	Y	2.75
Systems Analyst	1.02	4.27	2.81	3.61	3.17	Y	3.12
Factory Owner	1.02	3.81	3.49	2.88	3.71	n/a	
Supermarket Owner	1.21	3.51	3.27	1.96	3.45	n/a	
**SUMMARY: Quantitative cluster**		**4.40**	**3.05**	**2.64**	**3.05**	**44%**	**2.45**
		**Average Ratings by Skill**				O*NET	
**Verbal Cluster**	**Distance from Cluster Center**	**Math**	**English**	**Science**	**Spatial**	**STEM ^a^**	**Spatial** **Visualization ^b^**
Librarian	0.43	2.17	4.44	1.93	3.30	N	2.12
Secretary	0.51	2.66	4.00	1.80	3.00	N	2.12
Refrigerator Sales ^c^	0.69	2.45	3.63	1.94	2.70	N	2.12
Writer	0.78	1.79	4.87	1.94	2.86	N	2.38
Comedian	0.96	1.55	3.98	1.59	2.47	N	2.62
Lawyer	1.05	2.91	4.58	2.67	3.22	N	2.50
**SUMMARY: Verbal cluster**		**2.26**	**4.25**	**1.98**	**2.92**	**0%**	**2.31**
		**Average Ratings by Skill**				O*NET	
**Basic & Applied** **Science Cluster**	**Distance from Cluster Center**	**Math**	**English**	**Science**	**Spatial**	**STEM ^a^**	**Spatial** **Visualization ^b^**
Meteorologist	0.33	3.94	3.00	4.62	3.50	Y	2.62
Med Lab Tech ^c^	0.36	4.03	3.00	4.59	3.43	Y	2.88
Lab Tech ^c^	0.36	4.23	3.00	4.63	3.55	Y	3.00
Geologist	0.55	3.72	2.76	4.72	3.60	Y	3.00
Environ Scientist ^c^	0.56	3.61	3.14	4.78	3.62	Y	2.75
Space Scientist	0.59	4.42	2.98	4.72	4.00	See Meteorologist	
Automotive Engr ^c^	0.60	4.22	2.68	4.06	3.90	Y	3.50
Comp Scientist ^c^	0.62	4.48	2.94	4.11	3.48	Y	3.25
Astronomer	0.62	4.28	2.87	4.79	4.08	Y	3.00
Biologist	0.63	4.01	3.14	4.90	3.33	Y	3.12
Electrical Engr ^c^	0.64	4.61	2.87	4.51	3.81	Y	3.12
Surgeon	0.67	4.06	3.39	4.69	4.24	Y	n/a
Comp Hardware ^c^	0.68	4.57	2.83	4.36	3.43	Y	3.25
Comp Software ^c^	0.68	4.59	2.99	4.09	3.88	Y	n/a
Physicist	0.70	4.63	3.07	4.73	3.83	Y	3.62
Geographer	0.74	3.56	2.89	3.93	3.98	Y	2.88
Pediatrician	0.75	3.54	3.45	4.67	3.37	Y	2.25
Doctor	0.79	3.87	3.66	4.84	4.01	Y	2.50
Astronaut	0.80	4.43	3.37	4.65	4.30	n/a	
Civil Engr ^c^	0.82	4.61	3.21	4.31	4.27	Y	3.75
Aerospace Engr ^c^	0.84	4.60	3.07	4.73	4.24	Y	3.25
Nuclear Engr ^c^	0.86	4.74	2.93	4.84	3.80	Y	3.12
Nurse	0.87	3.38	3.46	4.25	3.30	Y	3.00
Chemist	0.88	4.53	2.75	4.87	3.29	Y	3.00
Vet	0.88	3.42	2.89	4.67	3.16	Y	2.88
Engineer	0.90	4.80	3.06	4.67	4.08	See engineer specialties	
Electrician	0.92	3.76	2.56	3.74	3.49	N	3.38
Architectural Engr ^c^	0.95	4.70	3.06	4.37	4.39	See Civil Engineer	
Food Scientist	0.98	3.63	2.76	4.57	2.90	Y	2.88
Physical Therapist	1.02	3.07	3.07	4.15	3.45	Y	2.88
Dentist	1.10	2.94	3.05	4.46	3.91	Y	3.25
Airplane Pilot	1.15	3.71	3.19	3.63	4.50	N	3.38
Dietician	1.19	3.44	2.98	4.19	2.71	Y	2.50
School Teacher	1.25	4.02	4.26	3.99	3.71	N	2.88
Landscape Architect	1.26	3.91	2.76	3.44	4.45	Y	4.00
Architect	1.33	4.60	2.98	3.70	4.68	Y	4.12
Nutritionist	1.35	3.32	2.98	4.33	2.57	See Dietician	
Psychologist	1.73	2.70	4.07	4.19	3.25	Y	2.12
**SUMMARY: Basic & Applied** **Science cluster**		**4.02**	**3.08**	**4.41**	**3.72**	**91%**	**3.07**
		**Average Ratings by Skill**				O*NET	
**Spatial Cluster**	**Distance from Cluster Center**	**Math**	**English**	**Science**	**Spatial**	**STEM ^a^**	**Spatial** **Visualization ^b^**
Florist	0.30	2.28	2.43	2.64	3.58	N	3.88
Plumber	0.44	2.64	2.18	2.63	3.36	N	3.20
Cook Restaurant ^c^	0.51	2.28	2.35	2.49	2.96	N	2.88
Rancher	0.51	2.22	2.01	2.46	3.21	See Farmer	
Gardener	0.65	2.19	1.98	2.78	3.60	N	3.00
Baker	0.68	2.73	2.31	2.69	2.96	N	2.88
Elevator Operator	0.68	2.46	2.44	2.29	2.77	n/a	
Telephone Installer	0.77	2.92	2.70	2.85	3.57	N	3.00
Construction Worker	0.78	3.04	2.22	2.38	3.80	N	2.88
Umpire	0.83	2.24	2.57	1.60	3.62	N	2.62
Police Officer	0.85	2.31	3.19	2.11	3.18	N	2.62
Pro Athlete ^c^	0.86	1.90	2.03	1.82	3.61	N	3.00
Hair Stylist	0.86	1.70	2.67	1.92	3.34	N	3.50
Welder	0.89	2.81	2.16	3.12	3.45	N	2.88
Clothing Designer	0.94	2.84	2.81	1.88	3.96	N	3.63
Truck Driver	1.00	1.95	2.03	1.59	3.28	N	3.00
Artist	1.10	2.11	2.66	1.97	4.40	N	4.00
Ship Captain	1.21	3.09	2.89	3.07	3.96	N	3.25
Manicurist	1.22	1.70	2.19	1.68	2.76	N	2.50
Ballet Dancer	1.28	1.64	1.85	1.60	3.82	N	2.75
Birth Attendant	1.29	2.30	2.90	3.53	3.09	Y	2.62
Auto Mechanic	1.34	3.21	2.41	3.25	4.05	N	3.38
Farmer	1.35	2.77	2.30	3.57	3.94	N	2.88
Dental Assistant	1.37	2.81	3.07	3.51	3.31	Y	2.88
Babysitter	1.37	1.66	2.85	1.66	2.63	N	2.75
Interior Decorator	1.39	3.00	2.84	2.02	4.56	N	4.00
Dishwasher ^c^	2.10	1.40	1.60	1.35	2.15	N	2.00
**SUMMARY: Spatial cluster**		**2.38**	**2.43**	**2.39**	**3.44**	**8%**	**3.04**

Note. Summary statistics for each job cluster are in bold at the end of the cluster listing. Cells marked n/a were those jobs that either lacked complete O*NET data or had no job that was a reasonable match. Some jobs we listed separately were grouped in the same job entry in O*NET; we did not repeat the O*NET data for the duplicates. Surgeon had five types listed on O*NET but all were highly specialized, except for the one labeled “other” with no details. ^a^ From “All STEM Occupations” (NCOD 2023e) by the U.S. Department of Labor, Employment and Training Administration (USDOL/ETA). Used under the CC BY 4.0 license. ^b^ From “Abilities—O*NET 27.3 Data Dictionary” (NCOD 2023a) by the U.S. Department of Labor, Employment and Training Administration (USDOL/ETA). Used under the CC BY 4.0 license. ^c^ Job title abbreviated for table display; see https://osf.io/6dby2 (accessed on 3 November 2023) https://osf.io/s3a9z?view_only=136665bc7f6d4a88965e475248151897 for verbatim job title used in the surveys.

**Table 3:** Means and standard deviations for self-concepts, job interest, job cultural stereotyping, and spatial performance by participant gender.

Survey Sections	Women		Men			
*Self-concepts by domain* (range 1–7)	*M*	*SD*	*M*	*SD*	*d*	*p*
Math	4.4	1.5	4.7	1.4	−0.35	.003
English	5.5	1.0	4.9	1.1	0.49	<.001
Science	4.5	1.5	4.9	1.4	−0.30	
Spatial	5.0	1.0	5.1	1.0	−0.11	
Athletics	4.8	1.4	5.4	1.3	−0.41	.001
Foreign Language	4.6	1.4	4.1	1.4	0.36	.003
						
*Job interest by cluster* (range 1–5)	*M*	*SD*	*M*	*SD*	*d*	*p*
Quantitative	1.5	0.7	1.8	0.8	−0.32	.002
Verbal ^a^	2.0	0.7	1.8	0.6	0.24	
Basic and Applied Science	1.8	0.6	2.0	0.7	−0.31	.010
Spatial	1.7	0.6	1.6	0.5	0.31	.011
						
*Job gender stereotyping* ^b^ *by cluster* (range 1–5)	*M*	*SD*	*M*	*SD*	*d*	*p*
Quantitative	2.6	0.5	2.7	0.4	−0.15	
Verbal ^c^	3.1	0.2	3.1	0.3	0.25	
Basic & Applied Science	2.6	0.3	2.7	0.3	−0.15	
Spatial ^d^	2.8	0.2	2.8	0.2	−0.11	
						
*Spatial performance* (range 0–15)	*M*	*SD*	*M*	*SD*	*d*	*p*
Water-level task score	8.7	4.1	10.2	4.4	−0.37	.001

Note. *N*s for the analyses were 138–142 women and 145–148 men. Ranges listed are possible scores on each measure. The directions of the scores for the measures are as follows: higher self-concept scores = more positive self-concepts; higher job interest scores = greater interest; stereotyping scores <3 = more masculine, >3 = more feminine, 3 = gender neutral; and higher water-level task scores = better spatial performance. The *p*-values reported are the significant ones based on adjusted values from follow-up comparisons using MANOVAs or from *t*-tests (full explanations in text). ^a^ Cronbach’s alpha = .58. ^b^ Beliefs about the culture’s gender stereotyping. ^c^ Cronbach’s alpha near zero. ^d^ Cronbach’s alpha = .52.

**Table 4:** Basic & applied science cluster job interest: No gender difference model.

Path	*B*	*SE*	*p*	*β*
Direct: Cultural gender stereotyping of basic & applied science jobs to spatial self-concept	−0.009	0.165	.956	−.003
Direct: Cultural gender stereotyping of basic & applied science jobs to basic & applied science job cluster interest	0.124	0.110	.258	.066
Direct: Spatial self-concept to basic & applied science job cluster interest	0.091	0.044	.039	.134
Direct: Spatial performance to spatial self-concept	0.079	0.015	<.001	.334
Direct: Spatial performance to basic & applied science job cluster interest	0.026	0.011	.015	.162
Indirect: Cultural gender stereotyping of basic & applied science jobs to spatial self-concept to basic & applied science job cluster interest	−0.001	0.016	.857	0
Indirect: Spatial performance to spatial self-concept to basic & applied science job cluster interest	0.007	0.024	.038	.045

**Table 5:** Quantitative cluster job interest: Gender difference model.

Path	*B*	*SE*	*p*	*β*
Direct: Cultural gender stereotyping of quantitative jobs to math self-concept				
Women	0.682	0.237	.004	.250
Men	0.264	0.288	.359	.079
Direct: Cultural gender stereotyping of quantitative jobs to quantitative cluster job interest				
Women	0.338	0.115	.003	.235
Men	−0.304	0.138	.028	−.174
Direct: Math self-concept to quantitative job cluster interest				
Women	0.168	0.031	<.001	.318
Men	0.168	0.031	<0.001	.322
Indirect: Cultural gender stereotyping of quantitative jobs to math self-concept to quantitative cluster job interest				
Women	0.115	0.039	.007	.080
Men	0.045	0.039	.391	.026

## Data Availability

The participant data and data dictionaries used in this research are available at https://osf.io/59qm2/ (accessed 3 November 2023). The O*NET data used to compare with our survey’s jobs are from the U.S. Department of Labor, Employment and Training Administration (USDOL/ETA), used under the CC BY 4.0 license. The comparisons are available at https://osf.io/6dby2/ (accessed 3 November 2023).

## Supplementary Materials

Supplementary Tables: Table S1. Correlations; Table S2. Correlations by participant gender, both at https://osf.io/874ew/ (accessed 3 November 2023); Job Skills Rating (JSR) Survey https://osf.io/7q6zh (accessed 3 November 2023); Self-Concept, Interest, Belief, and Spatial (SCIBS) survey: https://osf.io/fjt6m/ (accessed 3 November 2023); Job Skills Rating (JSR) Survey Jobs Compared to O*NET: https://osf.io/6dby2 (accessed 3 November 2023); Data Files and Dictionaries: https://osf.io/59qm2/ (accessed 3 November 2023).
